# Constructing a doxycycline-inducible system for an epithelial-to-mesenchymal transition model in MCF10A cells

**DOI:** 10.1242/bio.061790

**Published:** 2024-12-09

**Authors:** Yaxuan Sun, Xun Zhou, Xiaohui Hu

**Affiliations:** ^1^ Department of Pathology, School of Basic Medical Sciences, Anhui Medical University, Hefei 230032, China; ^2^ Department of Pathophysiology, School of Basic Medical Sciences, Anhui Medical University, Hefei 230032, China

**Keywords:** MCF10A, EMT, Cancer metastasis, TWIST1, Doxycycline inducible

## Abstract

Epithelial to mesenchymal transition (EMT) has been shown to play an essential role in the early stages of cancer cell invasion and metastasis. Inducible EMT models can initiate EMT in a controlled manner, thereby providing the opportunity to determine whether a cancer-associated gene influences cancer metastasis by triggering EMT. Moreover, different inducible EMT models enable the investigation of specific mechanisms of EMT modulation by various genes, facilitating a more precise understanding of how these genes influence cancer metastasis through the induction of EMT. Unfortunately, current inducible EMT models still present unmet needs. Therefore, we aimed to establish an inducible EMT model in MCF10A cells, a spontaneously immortalized human fibrocystic mammary cell line, by manipulating the expression of mouse Twist1 (mTwist1). In this study, we first compared the EMT induction capacity between human TWIST1 (hTWIST1) and mTwist1, and selected mTwist1 for further investigation. By monitoring the changes in epithelial and mesenchymal markers at different induction time points, we examined the EMT process in both polyclonal and monoclonal MCF10A cells that express doxycycline (DOX)-inducible mTwist1. Furthermore, our results showed that doxycycline-induced mTwist1 expression triggered EMT at a similar rate to TGFβ1-induced EMT in MCF10A cells. Additionally, this process was reversible upon DOX withdrawal. Thus, we have established a robust inducible EMT model in MCF10A cells, which can be used to further study cancer metastasis-driving genes.

## Teaser

**Summary:** A robust inducible epithelial to mesenchymal transition (EMT) model was established in MCF10A cells using doxycycline-induced mTwist1 expression. This model will facilitate researchers to investigate cancer metastasis-driving genes and their effects on Twist1-mediated EMT.

## INTRODUCTION

Epithelial to mesenchymal transition (EMT) plays essential roles in physiological conditions, such as embryo development, and pathological conditions, like cancer metastasis (Thiery et al., 2009). Although the significance of EMT in cancer metastasis was still a debated topic until recently, it has been demonstrated to contribute to cancer cell invasion and distal metastasis through accumulating evidence, ranging from direct *in vivo* imaging to mechanistic insights (Bakir et al., 2020; Wyckoff et al., 2007). During the transition, the cobblestone-like epithelial cells adopt an elongated spindle-like morphology by eliminating epithelial protein markers and acquiring mesenchymal protein markers, remodeling cell adhesion and the cytoskeleton. As a result, cancer cells dissociate from the original clusters to migrate and invade into blood or lymphatic vessels, initiating distal metastasis (Boyer et al., 1989). These mesenchymal cells undergo mesenchymal-to-epithelial transition (MET) at the distal site to facilitate tumor colonization, suggesting a dynamic and plastic regulatory network during EMT (Hugo et al., 2007). EMT is induced by a number of signaling pathways, transcription factors, and non-coding RNAs (De Craene and Berx, 2013). The signaling pathways involved in the induction of EMT, include EGF, FGF, HGF, TGFβ, BMPs, WNTs, and Notch signaling (Barrallo-Gimeno and Nieto, 2005). Downstream of these signaling pathways, transcription factors trigger EMT-associated transcriptomic changes by decreasing the expression of epithelial markers and increasing the expression of mesenchymal markers. The nuclear transcription factors TWIST, SNAIL, and ZEB family members are the most widely studied in relation to EMT induction (De Craene and Berx, 2013).

A group of genes has been identified that influence cancer metastasis by modulating EMT. However, EMT is not usually triggered immediately after manipulating the expression of these genes, such as Hic-5, hnRNPM, hnRNPF, and AKAP8. Instead, their impact on EMT could be observed in the inducible systems (Hu et al., 2020; Huang et al., 2017; Xu et al., 2014; Pignatelli et al., 2012). Currently, several inducible EMT systems are available. TGFβ1, as one of the well-known cytokines, can induce EMT in many types of cells, including HMLE cells (Mani et al., 2008), MCF10A cells (Antón-García et al., 2023), and lung epithelial H358 cells (Thomson et al., 2011). Another cytokine, TNF-α, has been reported to induce EMT in HCT116 cells within 4 days (Wang et al., 2013). Among the different inducible EMT models, approximately 60% of differentially expressed EMT-associated genes show cell-type-specific patterns, emphasizing the need to investigate the function of EMT-associated genes in multiple EMT models (Knutsen et al., 2023). While TGFβ1-induced EMT models have been established in multiple cell lines, EMT transcription factor (EMT-TF)-inducible EMT models have been mainly established by Dr. Robert A. Weinberg's group in HMLE cells (Mani et al., 2008). HMLE cells are derived from multipotent mammary stem cells (Elenbaas et al., 2001), while the MCF10A cell line was originally isolated from human fibrocystic mammary tissue (Soule et al., 1990), and has been used to develop various model systems for studying the tumorigenic transformation from normal breast epithelium to malignancy (Puleo and Polyak, 2021). These factors prompted us to seek an EMT-TF-mediated EMT-inducing system in MCF10A cells.

Twist1, as a master regulator of EMT, is critical for both physiological and pathological processes (Thiery et al., 2009). The human and mouse Twist1 genes were originally cloned by Dr. Fabienne Perrin-Schmitt's lab and have been made available to scientists worldwide to perform Twist1 functional research (Bourgeois et al., 1996; Wolf et al., 1991). Due to the high similarity between mouse and human Twist1 (mTwist1 and hTWIST1, respectively), mTwist1 has been used in vast majority of research studies to induce EMT induction in human cells (Hu et al., 2014; Yang et al., 2004). However, there is no direct evidence comparing their ability to induce EMT. In this study, we first cloned both mTwist1 and hTWIST1 cDNA to overexpression vectors. We then constructed MCF10A cell lines that stably express mTwist1 or hTWIST1 and compared their potential to induce EMT in MCF10A cells. Additionally, we cloned mTwist1 to a pLVX-TetOne vector to create a DOX-inducible system for EMT in MCF10A cells. These cells exhibited a gradual EMT process upon DOX treatment, although they slightly reverted to the epithelial state after 18 days of induction in polyclonal MCF10A cells. This issue can be addressed by isolating monoclonal MCF10A cells that stably express Twist1 for EMT induction. The speed of EMT induction in our monoclonal MCF10A cell was comparable to that of TGFβ1-induced EMT in MCF10A cells. Furthermore, MCF10A cells undergoing EMT can revert to an epithelial cell state when DOX is withdrawn from the culture, indicating the full process of EMT and MET, making this model suitable for cancer metastasis studies.

## RESULTS

### Cloning of hTWIST1 and mTwist1

**Fig. 1.**
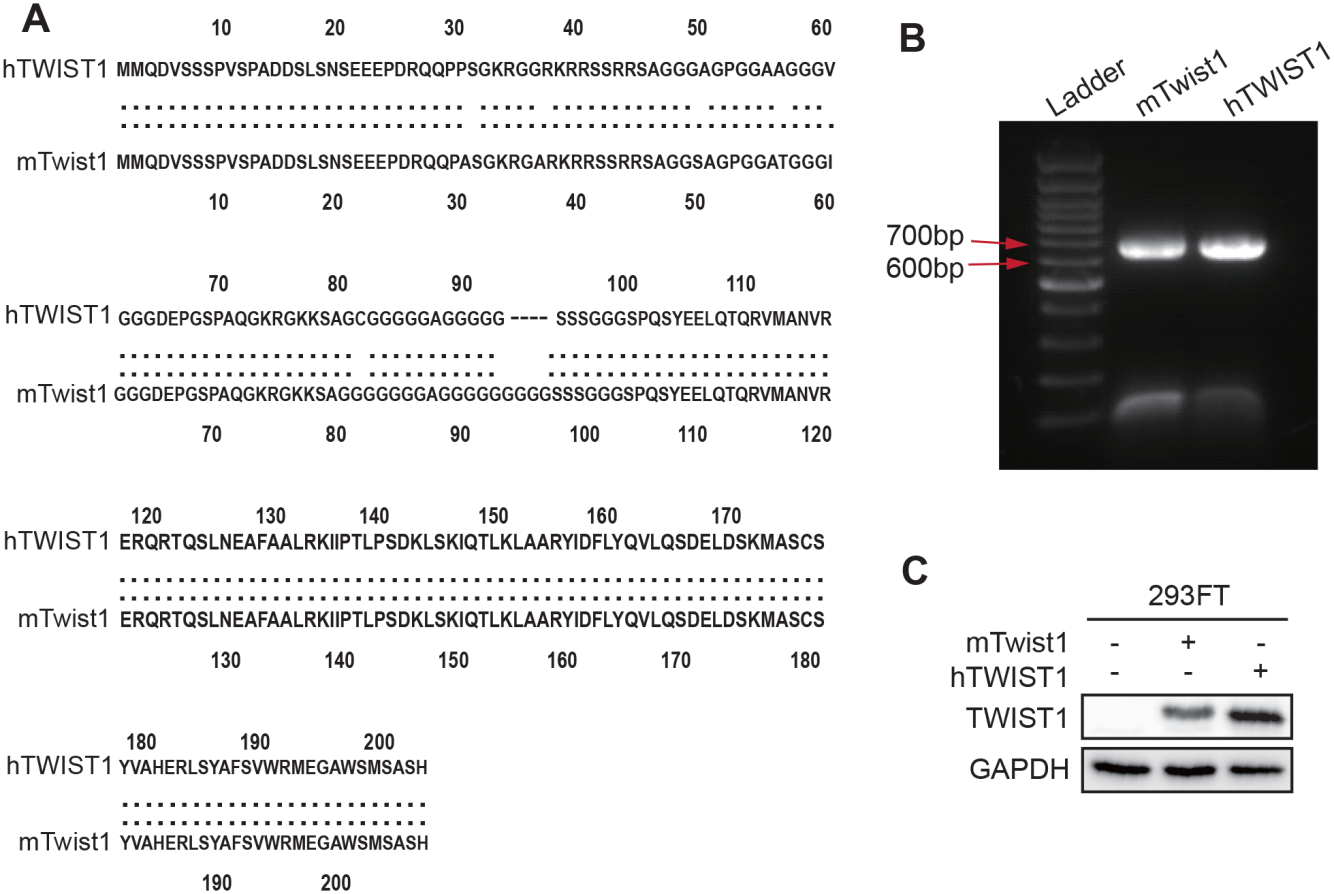
**Cloning mTwist1 and hTWIST1 cDNA.** (A) Protein sequence comparation between mTwist1 and hTWIST1 using Lalign. (B) PCR amplification of *mTwist1* and *hTWIST1* cDNA. (C) Western blot detection of hTWIST1 and mTwist1 expression in 293FT cells transiently transfected with pLenti-mTwist1 or pLenti-hTWIST1.

The current EMT induction in human cells by Twist1 largely stems from mouse cDNA. Since the protein sequences of hTWIST1 and mTwist1 share over 95% identity (Fig. 1A), mTwist1 was considered to have identical activity to hTWIST1. Although this high similarity suggests that both proteins have the potential to induce EMT in a similar manner, no published data directly compares their EMT induction abilities in parallel. To address this, we amplified both *hTWIST1* and *mTwist1* cDNA sequences (Fig. 1B) and cloned them into the pLenti-CMV-MCS-BLAST vector (Fig. S1A,B). The two plasmids were then transiently transfected into 293FT cells to verify their expression. Both proteins were successfully expressed in 293FT cells at expected size (Fig. 1C). However, we observed that the band density of mTwist1 appeared weaker than that of hTWIST1, which might be due to the immunoblotting antibody being produced from hTWIST1 peptide and recognizing fewer epitopes on mTwist1. These two vectors will be used to further functional studies.

### hTWIST1 and mTwist1 showed identical ability to induce EMT in MCF10A cells

**Fig. 2.**
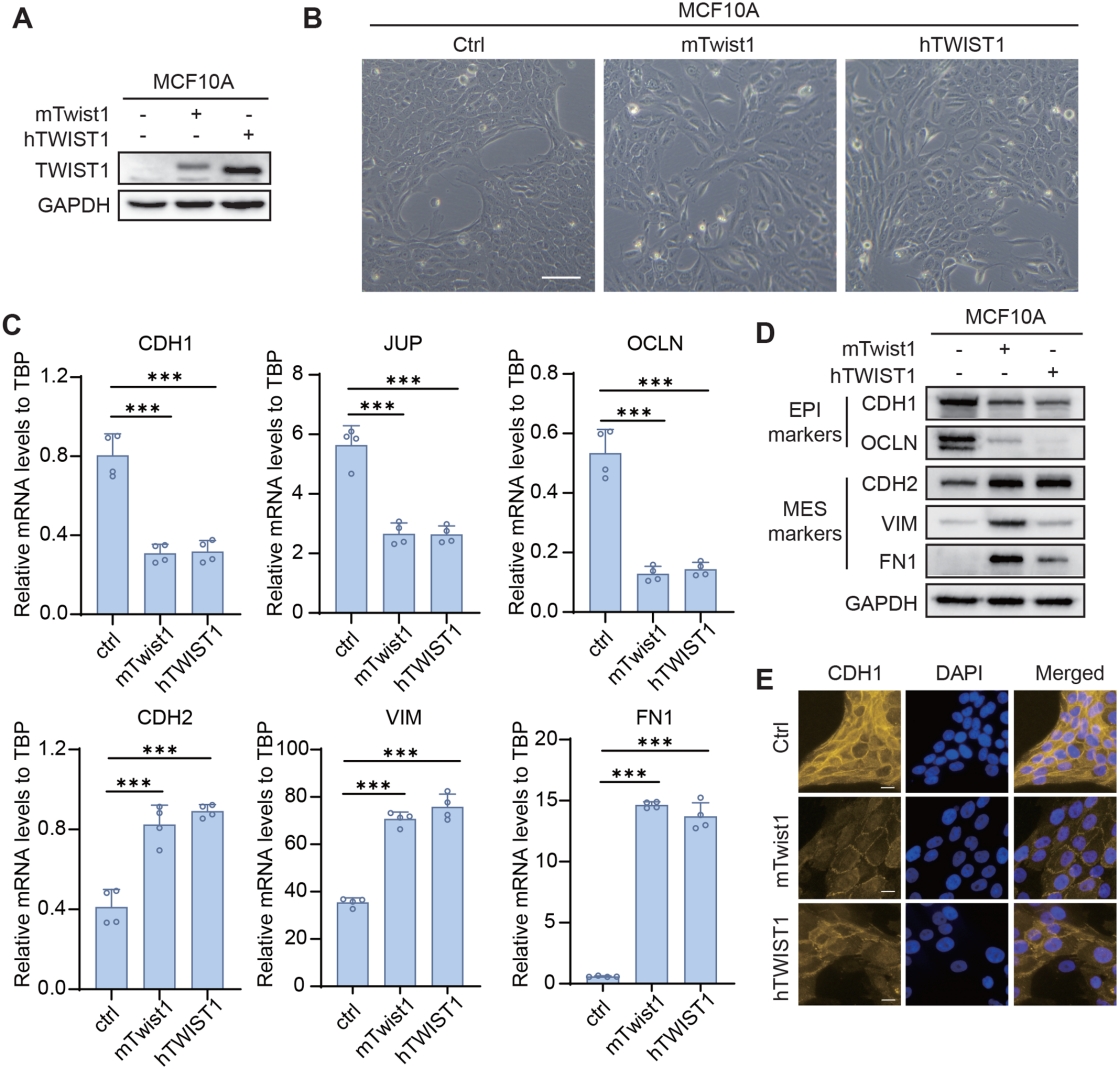
**mTwist1 and hTWIST1 exhibit comparable EMT induction abilities.** (A) Detection of mTwist1 and hTWIST1 expression in sMCF10A-mTwist1 and sMCF10A-hTWIST1 cells. (B) Representative images showing the morphology of control MCF10A (Ctrl), sMCF10A-mTwist1 and sMCF10A-hTWIST1 cells. Scale bar: 100 μm. (C) Expression of EMT markers in control (ctrl), sMCF10A-mTwist1 and sMCF10A-hTWIST1 cells was detected by qRT-PCR. Data are presented as mean±s.d. *P*-values were calculated by two-tailed unpaired Student's *t*-test. *N*=4, ***P<0.001. (D) Western blotting showing EMT marker expression in the same cells. (E) Expression of CDH1 in ctrl, sMCF10A-mTwist1 and sMCF10A-hTWIST1 cells analyzed by immunofluorescence. Scale bars: 20 μm.

To compare the EMT induction ability of mTwist1 and hTWIST1, we generated MCF10A cell lines that stably express either mTwist1 or hTWIST1 (Fig. 2A). The cell lines were termed as sMCF10A-mTwist1 and sMCF10A-hTWIST1, respectively. After several passages, both cell lines exhibited a spindle-like morphology (Fig. 2B), suggesting a transition to mesenchymal phenotype. This was further validated by changes in the expression of EMT markers, as assessed by qRT-PCR (Fig. 2C). Both sMCF10A-mTwist1 and sMCF10A-hTWIST1 showed a comparable decrease in epithelial markers, such as CDH1, JUP and OCLN, and an increase in mesenchymal markers, such as CDH2, VIM and FN1. Western blotting presented similar trends (Fig. 2D), although there were some discrepancies in mesenchymal markers like VIM and FN1. Since qRT-PCR detected equivalent levels of these markers in both mTwist1 and hTWIST1 cells, we speculated the variation might result from differences in western blotting detection. As CDH1 expression on the cell surface is a marker of epithelial status, we also performed the immunofluorescence assay for CDH1 in control and Twist1-expression cells. Both mTwist1 and hTWIST1 cells showed comparable attenuation of CDH1 signaling (Fig. 2E). These results suggested that mTwist1 and hTWIST1 have comparable abilities to induce EMT in MCF10A cells. To be consistent with the current EMT induction models, we choose mTwist1 for subsequent research.

### Doxycycline induces a gradual EMT in MCF10A cells stably expressing pLVX-TetOne-mTwist1

**Fig. 3.**
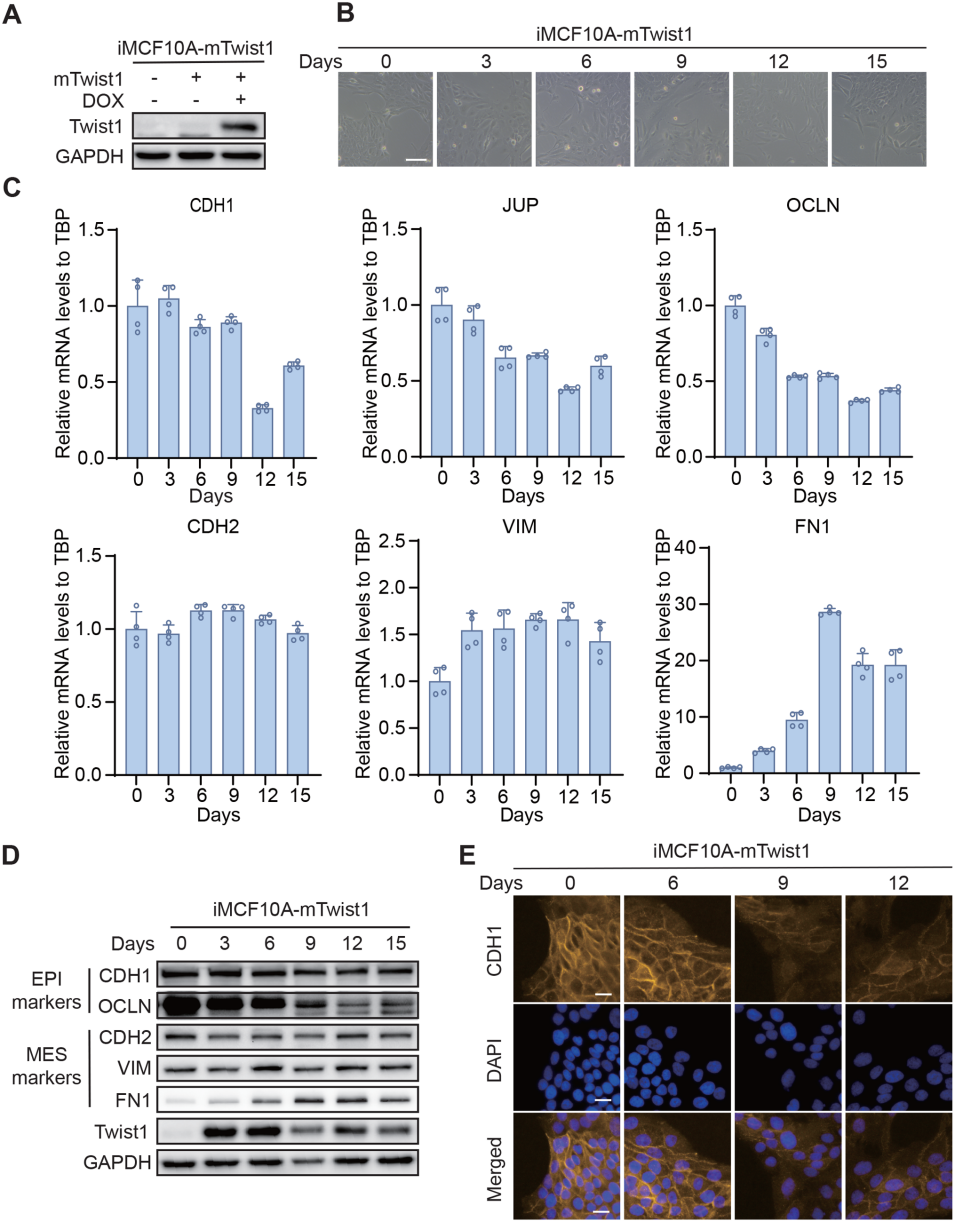
**DOX induces a gradual EMT in MCF10A cells stably expressing pLVX-TetOne-mTwist1.** (A) Expression of mTwist1 can be induced by DOX. (B) Representative Images showing morphological changes of iMCF10A-mTwist1 cells over time with DOX induction at 1 μg/ml. Scale bar: 100 μm. (C) Expression of EMT markers in cells collected at indicated time points, analyzed by qRT-PCR. Data are presented as mean±s.d., *N*=4. (D) Western blotting analysis of EMT marker expression at the same time points. (E) Expression of CDH1 in cells collected at different time points, analyzed by immunofluorescence. Scale bars: 20 μm.

To construct an inducible EMT system, we subcloned *mTwist1* cDNA into the DOX-inducible pLVX-TetOne lentiviral expression vector (Fig. S2A). The construct was then transduced into MCF10A cells (iMCF10A-mTwist1) and mTwist1 expression could be induced by DOX treatment (Fig. 3A). To induce EMT, iMCF10A-mTwist1 cells were split every 3 days and DOX was added during each cell passage (Fig. S2B). Over time, cell morphology transitioned to a spindle-like appearance, indicating the loss of tight cell-cell connections and mesenchymal transition, especially by day 12 (Fig. 3B). Samples from different time points were collected to examine the dynamic alterations in EMT marker expression (Fig. S2B). We observed a gradual increase in mesenchymal markers and a gradual decrease of epithelial markers, both at mRNA and protein levels (Fig. 3C,D), indicating a gradual EMT process. This was further confirmed by the immunofluorescence staining of CDH1, which showed decreased expression of CDH1 on the cell surface with DOX induction (Fig. 3E). However, a complete turnover of EMT markers was not observed, even by day 15. Additionally, the decrease in epithelial markers and increase in mesenchymal markers plateaued at day 15, suggesting that EMT induction might be halted at this point. We also noted that mTwist1 expression decreased over time, even in the presence of DOX (Fig. 3D), suggesting negative selection of the mTwist1-expression population in the polyclonal iMCF10A-mTwist1 cells.

### Mesenchymal cell state can be reversed in polyclonal MCF10A cells expressing mTwist1

**Fig. 4.**
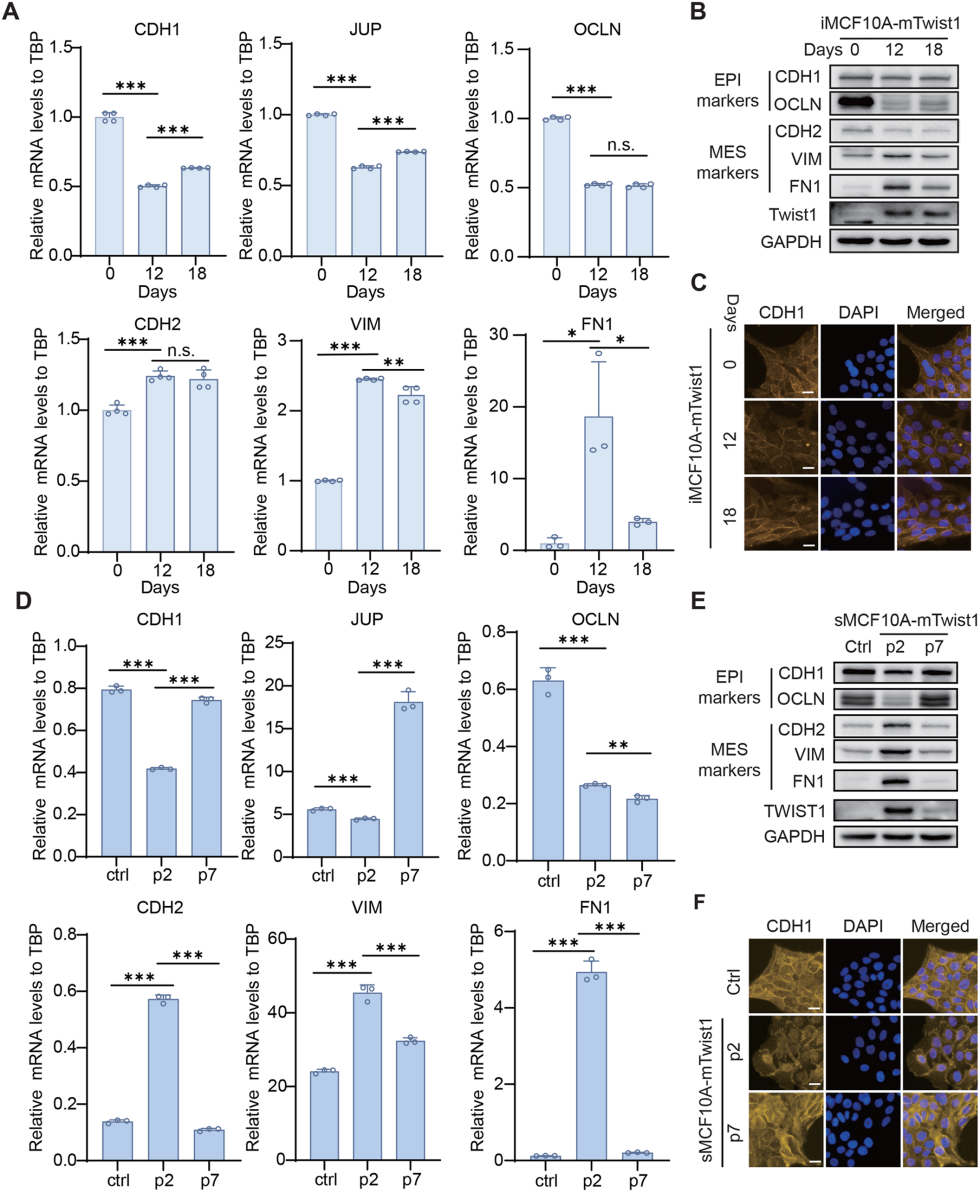
**The mesenchymal transition can be reversed in polyclonal MCF10A cells expressing mTwist1.** The expression levels of EMT markers in iMCF10A-mTwist1 cells treated with DOX for 0, 12, and 18 days were detected by qRT-PCR (A) and western blotting (B). (C) Representative images showing the expression of CDH1 in iMCF10A-mTwist1 cells treated with DOX for 0, 12, and 18 days. The expression levels of EMT markers in ctrl and sMCF10A-mTwist1 cells collected at p2 and p7 were analyzed by qRT-PCR (D) and western blotting (E). (F) Immunofluorescence images showing CDH1 expression in Ctrl and sMCF10A-mTwist1 cells collected at p2 and p7. Scale bars in C and F: 20 μm. qRT-PCR data are presented as mean±s.d., *N*=4 for A and *N*=3 for D. *P<0.05, **P<0.01, ***P<0.001; n.s., not significant.

To test whether the DOX inducible EMT system is stable in polyclonal iMCF10A-mTwist1 cells, we continued DOX treatment for several more days. Compared to the EMT marker expression at day 12, cells at day 18 showed partial restoration of epithelial markers and a slight decrease in mesenchymal markers, indicating a retreat of EMT (Fig. 4A,B and Fig. S3A). Re-expression of CDH1 on the cell surface further confirmed the recovery of an epithelial phenotype (Fig. 4C). These results suggest that EMT can be reversed in polyclonal iMCF10A-mTwist1 cells, even in the presence of DOX induction.

We also tested whether EMT could retreat in sMCF10A-mTwist1 cells. After culturing sMCF10A-mTwist1 cells for several more passages, we collected them to assess EMT marker expression. Interestingly, by passage 7 (p7), the mesenchymal phenotype reverted to an epithelial phenotype in sMCF10A-mTwist1 cells (Fig. S3B). Epithelial markers increased in p7 cells, while mesenchymal markers decreased (Fig. 4D,E). Consistently, p7 cells also restored CDH1 expression on the cell surface (Fig. 4F). The downregulation of mTwist1 expression in both iMCF10A-mTwist1 and sMCF10A-mTwist1 cells (Fig. 4B,E) suggests that the retreat of EMT may be due to negative selection, where mTwist1-expression population loses its proliferative advantage and is eliminated over time. Interestingly, the recovery of the epithelial marker OCLN was not as significant in both stable and inducible EMT systems, suggesting OCLN alteration is stable once cells have transitioned to mesenchymal state.

### Monoclonal iMCF10A-mTwist1 cells showed steady epithelial to mesenchymal transition

**Fig. 5.**
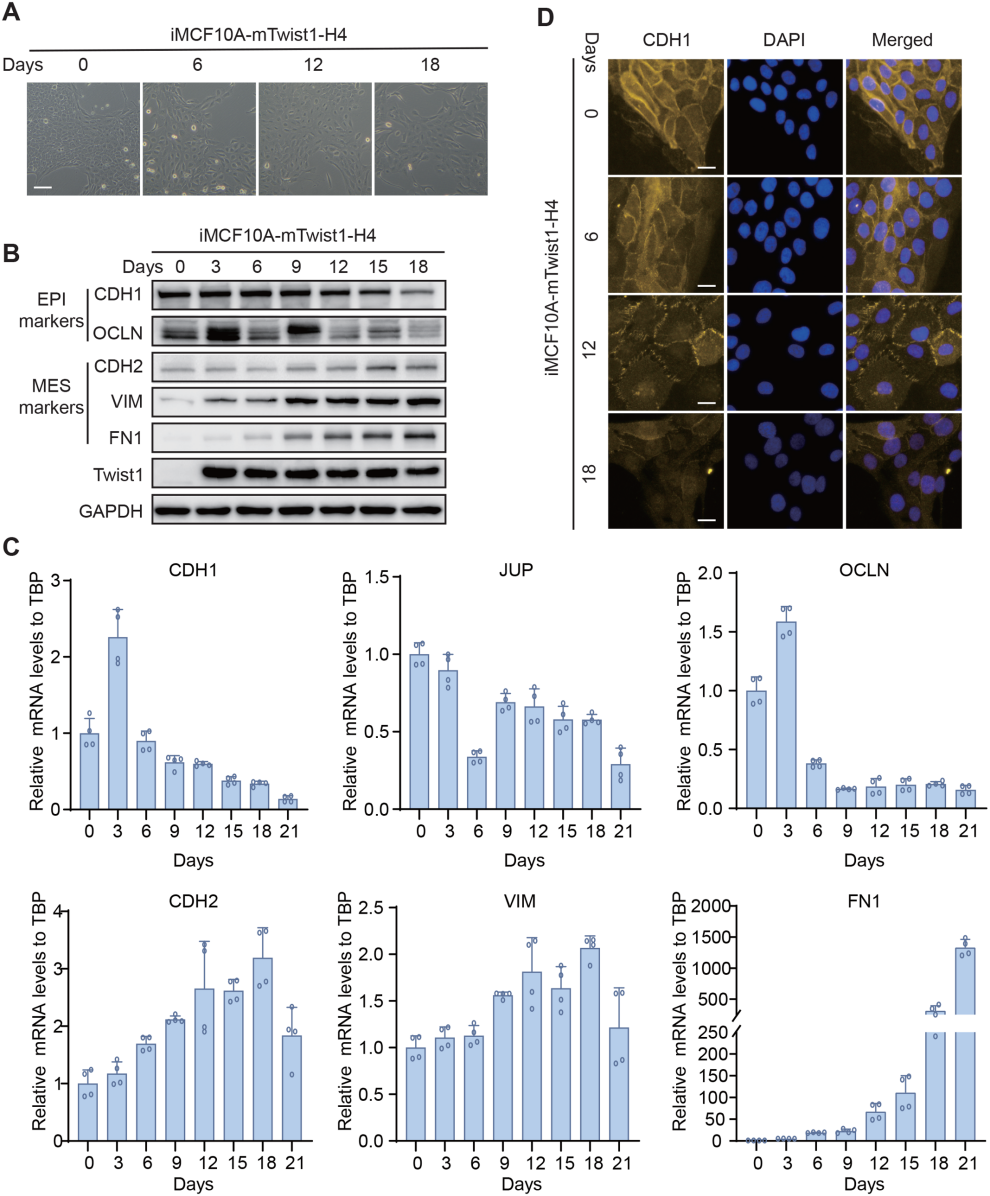
**Monoclonal iMCF10A-mTwist1 cells show a stable EMT.** (A) Representative images showing morphological changes in monoclonal iMCF10A-mTwist1-H4 cells treated with DOX on days 0, 6, 12, and 18. Scale bar: 100 μm. (B) The expression of EMT markers of clone H4, detected by western blotting at indicated time points. (C) qRT-PCR analysis of EMT marker expression in clone H4 at indicated points. Data are presented as mean±s.d., *N*=4. (D) Expression of CDH1 in clone H4, analyzed by immunofluorescence at indicated time points. Scale bars: 20 μm.

To create a more stable inducible EMT model and overcome the retreat of EMT observed in polyclonal cells, we isolated monoclonal iMCF10A-mTwist1 cells via blasticidin-S selection. The clones that expressed relative high levels of mTwist1 after DOX treatment were selected for further EMT induction assays (Fig. S4A). Upon DOX treatment of clone H4, we observed a steady morphological transition from epithelial to mesenchymal status over time (Fig. 5A). By examining the expression of EMT markers at different time points using qRT-PCR and western blotting, we observed a steady decrease in epithelial markers and an increase in mesenchymal markers, even at day 21 (Fig. 5B,C). Immunofluorescence staining of CDH1 on the cell membrane showed a similar trend (Fig. 5D). Furthermore, we induced EMT in two other monoclonal iMCF10A-mTwist1 cells, A11 and A7, both of which displayed a gradual and steady EMT process (Figs S4 and S5). As expected, mTwist1 expression remained stable in all three clones (Fig. 5B; Figs S4C and S5B). Thus, monoclonal iMCF10A-mTwist1 cells overcame the retreat of EMT observed in the polyclonal cells.

Currently, the inducible EMT model in MCF10A cells primarily relies on TGFβ1 induction, with variations in the dosage and duration of TGFβ1 treatment across different groups (Antón-García et al., 2023; Brown et al., 2011; Du et al., 2016). To compare the characteristics of EMT induction between our Twist1 model and the TGFβ1 model, we treated MCF10A cells with 5 ng/ml TGFβ1 for over two weeks (Fig. S6A) and collected cells at indicated time points to assess the alterations of EMT markers (Fig. S6B-D). Our Twist1 model showed a similar EMT induction speed to TGFβ1 model when comparing the switching of EMT markers at corresponding time points. However, their extent of expression changes showed slight differences in OCLN and FN1. The Twist1 model exhibited a greater decrease in OCLN and an increase in FN1, suggesting that the two models induce EMT through different mechanisms. Since TGFβ1 and Twist1 initiate EMT at the top or bottom of the signaling cascade, respectively, these two models can be utilized simultaneously to study at which stage a given gene interferes with EMT, enriching our understanding of cancer metastasis mechanisms. Taken together, our monoclonal iMCF10A-mTwist1 cell line provides a stable and comprehensive EMT induction model to complement future research.

### iMCF10A-mTwist1 cells undergoing EMT reverted to an epithelial status after DOX withdrawal

**Fig. 6.**
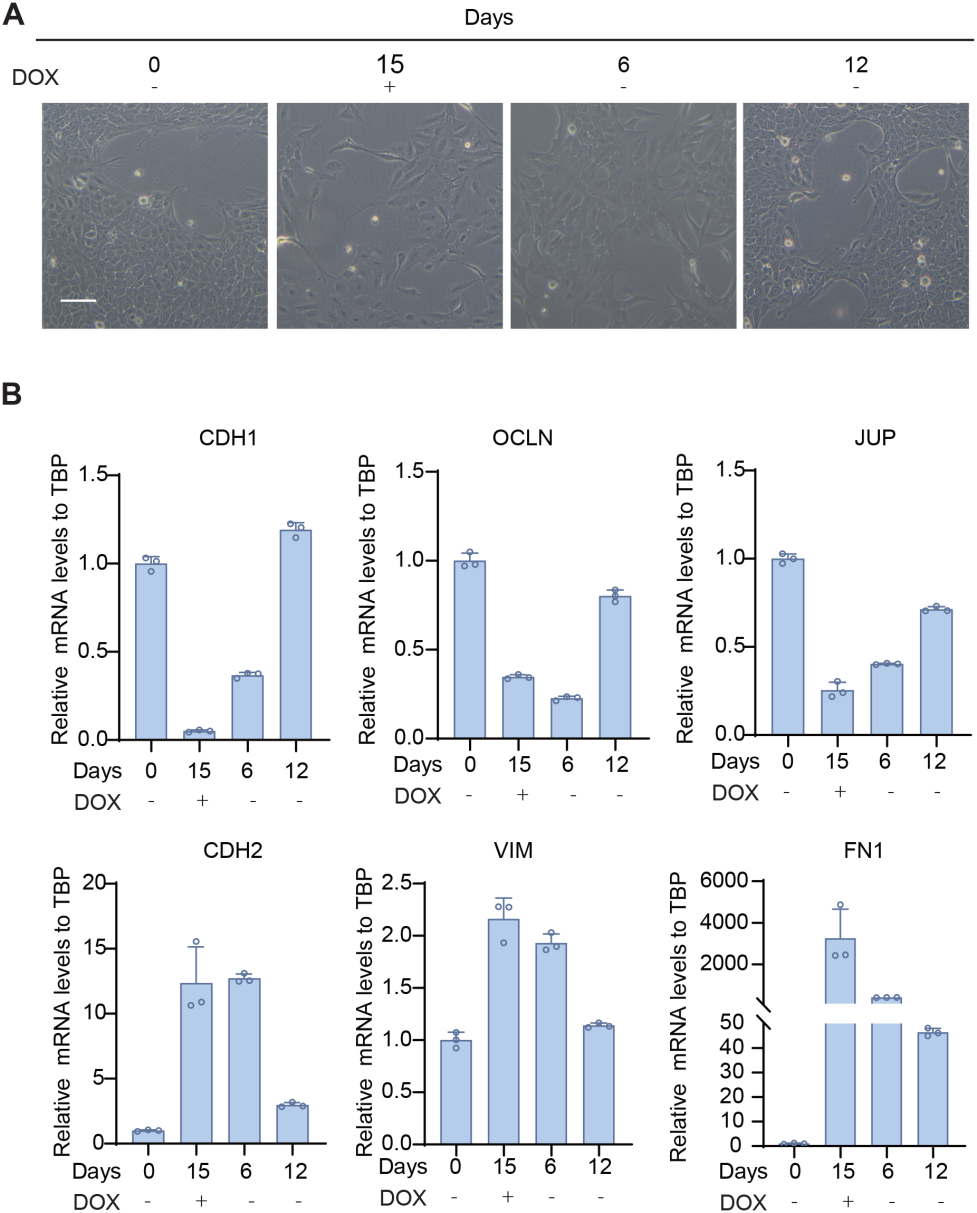
**EMT is reversible after DOX withdrawal in iMCF10A-mTwist1-H4 cells.** (A) Representative images showing morphological changes in iMCF10A-mTwist1-H4 treated with DOX on days 0 and 15, following DOX withdrawal on days 6 and 12. Scale bar: 100 μm. (B) qRT-PCR analysis of EMT marker expression at indicated time points. Data are presented as mean±s.d., *N*=3.

As a flexible induction system, it's critical to be reversible after the withdrawal of induction factors. To verify this in our system, we cultured the monoclonal iMCF10A-mTwist1-H4 cells that had undergone EMT in the absence of DOX for several more passages. After 6 days of culture, the cells began to cluster and revert to an epithelial cell state, as observed through changes in cell morphology and expression of EMT markers (Fig. 6A,B). Their morphology completely recovered to the cobblestone-like epithelial MCF10A cells after 12 days culture without DOX induction (Fig. 6A). The expression of EMT markers, as tested by qRT-PCR, showed a completed shift back to the epithelial cell state, with an increase in epithelial markers and a decrease in mesenchymal markers after 12 days culture without DOX (Fig. 6B). These results confirmed that our DOX inducible system for EMT is reversible.

## DISCUSSION

The expression of genes involved in EMT is regulated by a group of transcription factors, including the ZEB family (ZEB1 and ZEB2), the Snail family (Snail1, Snail2, Snail3), and the Twist family (Twist1, Twist2). The activities and expression levels of these EMT-TFs are controlled by EMT signal pathways and miRNAs during EMT process (Sánchez-Tilló et al., 2012). These EMT-TFs bind to the promoter regions of target genes, downregulating epithelial markers and upregulating mesenchymal markers, ultimately triggering EMT process. The ZEB family, being relatively large, contrasts with the smaller Snail and Twist families, which are easier to manipulate in cells (Sánchez-Tilló et al., 2012). Both Snail and Twist were used by Dr. Robert Weinberg's lab to establish the 4-OHT-inducible EMT system in HMLE cells (Mani et al., 2008). Among these, Snail seems to primarily repress the expression of epithelial markers, such as E-cadherin, whereas Twist not only suppresses the epithelial markers expression but also directly activates mesenchymal markers, such as N-cadherin (de Herreros et al., 2010; Alexander et al., 2006). Twist has been widely implicated in cancer metastasis (Ang et al., 2023), making it a promising candidate for EMT induction in various cell lines.

Twist1 and Twist2 share 66% protein sequence identity and function non-redundantly (Franco et al., 2011). Both proteins are classified as EMT-TFs and are frequently activated during carcinogenesis, with the majority of research focusing on the role of Twist1 in EMT and cancer metastasis. Based on this, we select Twist1 to construct the inducible EMT system in MCF10A cells.

Twist1 sequence is highly conserved across species. For example, human and mouse Twist1 protein share over 95% similarity (Fig. 1A). This high degree of similarity is one reason why the current EMT models using Twist1 are primarily based on mouse cDNA. To systematically compare the EMT induction efficiency of mTwist1 and hTWIST1, we cloned both cDNAs and stably expressed them in MCF10A cells. The two proteins showed comparable abilities to induce EMT in MCF10A cells, including changes in cell morphology and the switching of EMT marker expression. These results suggested that the slight difference in protein sequences does not significantly affect the binding affinity of TWIST1 to EMT initiation genes or its transcription activation efficiency. mTwist1 has been validated to induce EMT in multiple cell lines, including the kidney epithelial cells MDCK, human mammary epithelial cells (HMECs) and MCF10A, indicating its broad application of mTwist1 in EMT models (Hu et al., 2014; Yang et al., 2004). Therefore, we proceeded with the inducible EMT model using mTwist1.

To induce mTwist1 expression, we cloned mTwist1 into a Tet-On vector, pLVX-TetOne-blast. mTwist1 expression was only induced after DOX treatment, and no mTwist1 expression was detectable in the absence of DOX, ensuring tightly controlled EMT induction (Fig. 3A). Several studies have reported the inhibitory effect of DOX on EMT (Zhang et al., 2017; Xi et al., 2014; Meng et al., 2014). However, these studies used DOX concentrations up to 5 μg/ml to reverse EMT, while we treated iMCF10A-mTwist1 cells with only 1 μg/ml DOX to induce EMT, a concentration well below that which inhibits EMT. Additionally, Herr et al. reported that DOX had no effect on B-RafV600E-induced EMT in 3D cultures of MCF10A cells or on the invasive phenotype (Herr et al., 2011). These findings further support the notion that DOX is an effective agent for inducing target gene expression without interfering with EMT process at reasonable concentrations. However, we also observed that MCF10A cells proliferated very slowly and entered a quiescent state with prolonged DOX induction (i.e. over 21 days), which may be explained by the fact that EMT contributes to the acquisition of stem-like properties in cancer cells (Weidenfeld and Barkan, 2018). Although MCF10A are derived from human fibrocystic mammary tissue, further studies are necessary to verify whether these cells acquire a stemness signature during EMT. In addition, our study has not investigated the rate of EMT induction with DOX treatment at different concentrations in MCF10A cells, an assay that could help identify the optimal conditions for EMT induction.

During the EMT induction in polyclonal iMCF10A-mTwist cells, we did not observe a full transition to the mesenchymal phenotype, indicating the establishment of a partial EMT model. Moreover, we noticed a reversal expression of EMT markers by day 18 of DOX treatment. This issue was resolved when we isolated monoclonal iMCF10A-mTwist cells for EMT induction. Based on evidence showing that mTwist1 expression decreased over time in polyclonal cell line, we concluded that incomplete and reversed EMT in polyclonal cells was likely due to clonal selection of low mTwist1 expression cells. These results suggest that the efficiency of EMT induction can be optimized by isolating monoclonal cells, at least in MCF10A cells. This also provides a valuable insight for optimizing Twist1-mediated EMT induction in other cell lines.

In conclusion, we established an effective EMT induction model in MCF10A cells by isolating monoclonal cells that stably express mTwist1 in a DOX-inducible vector. This EMT system provides a robust model to investigate cancer metastasis-driving genes and their effects on Twist1-mediated EMT.

## MATERIALS AND METHODS

### Cell cultures

The MCF10A cell line was originated from ATCC and was maintained in DMEM/F12 medium (Procell, PM150310) supplemented with 5% horse serum (Gibco, 16050122), 20 ng/ml human epidermal growth factor (Novoprotein, C029), 0.5 μg/ml hydrocortisone (APExBIO, B1951), 10 μg/ml human recombinant insulin (Novoprotein, NC005), 0.1 μg/ml cholera toxin (Sigma, C8052), and penicillin/streptomycin (Beyotime, C0222). The 293FT cell line was a gift from Dr. Shoudong Ye's lab (Anhui University, China) and was cultured in DMEM high glucose (Gibco, 12800-017) supplemented with 10% FBS (Procell, 164210) and penicillin/streptomycin (Beyotime, C0222). Cell lines were tested for mycoplasma contamination routinely. All cells were maintained in humidified incubator at 37°C with 5% CO_2_.

### Plasmids construction

The open reading frames (ORFs) of mTwist1 and hTWIST1 were amplified from cDNA of H9C2 cells and MDA-MB-231 cells, respectively. The ORFs were ligated into pLenti-CMV-MCS-BLAST or pLVX-TetOne-BLAST backbone (HedgehogBio Science and Technology Ltd, Shanghai, China). All the insertions were verified by sequencing at Sangon Biotech (Shanghai, China). The primers used are listed in Table S1.

### Cell line generation and EMT induction

To generate MCF10A cell line that stably expressing mTwist1 or hTWIST1, pLenti-mTwist1 or pLenti-hTWIST1 plasmids were packaged with pSPAX2 and pMDG in 293FT cells to produce lentivirus. MCF10A cells were then plated and infected with the lentivirus containing mTwist1 or hTWIST1 and selected using blasticidin-S (Beyotime, ST018) for at least two passages to eliminate cells without the target plasmids. MCF10A cells infected with lentivirus containing the empty vector were used as controls. The cell lines stably expressing mTwist1 or hTWIST1 were termed sMCF10A-mTwist1 or sMCF10A-hTWIST1, respectively. MCF10A cells expressing DOX-inducible mTwist1 were generated similarly using the pLVX-TetOne-mTwist1 plasmid. The cell lines were termed iMCF10A-mTwist1.

To induce EMT, iMCF10A-mTwist1 cells were split every 3 days at a density of 3.5×10^5^ cells per 6 cm dish in the presence of 1 μg/ml DOX (Beyotime, ST039A) until the indicated time points. An additional dish was prepared at each passage to collect samples for EMT marker detection. To induce EMT with TGFβ1 in MCF10A cells, cells were split every 3 days at the density of 3.0×10^5^ cells per 6 cm dish in the presence of 5 ng/ml TGFβ1 (Novoprotein, CA59) until the indicated time points indicated. To isolate the monoclonal iMCF10A-mTwist1 cells, the cells were plated in a 96-well plate at a density of fewer than one cell per well and maintained in 10 μg/ml blasticidin-S until clones grew to a reasonable size. The clones were expanded and tested for the expression of mTwist1 to select positive ones.

### RNA extraction and quantitative real-time PCR (qRT-PCR)

Total RNA was extracted with Total RNA Extraction Reagent (Vazyme, R401-01) following the manufacturer's instructions. 1 μg of total RNA was reverse-transcribed using the First Strand cDNA Synthesis Kit (YEASEN, 11139ES10). To test the expression levels of target gene, qRT-PCR was performed with 0.5 μl of cDNA, 2×SYBR Green Master Mix (YEASEN, 11201ES08), and specific primers on a Bio-Rad CFX connect optics module. Relative mRNA levels were calculated using the 2^−ΔΔCt^ value by normalizing to TBP. The sequences of the qRT-PCR primers are listed in Table S1.

### Western blotting

Cells were collected and lysed in RIPA buffer containing freshly added protease inhibitors (Proteintech, PR20016) and phosphatase inhibitors (Proteintech, PR20015) on ice. Lysates were clarified by centrifuge at 12,000 rpm for 10 min at 4°C. Protein concentration was measured using BCA kit (Beyotime, P0010). Equal amounts of lysate were mixed with SDS loading buffer and boiled for 6 min. For each lane, 20 μg of protein was loaded onto a 10% SDS–PAGE gel, along with pre-stained protein markers (Proteintech, PL00001). After gel electrophoresis, proteins were transferred onto a methanol-activated PVDF membrane at 100 V for 2 h on ice. Membranes were blocked with 5% non-fat milk for 1 h at room temperature (RT), followed by overnight incubation with primary antibodies at 4°C. The primary antibodies and their dilution ratios are listed in Table S2. The next day, membranes were washed three times with TBST and incubated with HRP-conjugated secondary antibody for 1 h at RT. Membranes were washed four times with TBST and subjected to ECL luminescence detection using a ChemiDoc Imaging System from Bio-Rad.

### Immunofluorescence assay

Cells were cultured on tissue culture-treated glass slides. After removal, cells were fixed in 4% paraformaldehyde for 10 min, followed by three washes with PBS. Cells were then permeabilized with 0.2% Triton X-100 for 3 min, blocked with 2% BSA (Beyotime, 9048-46-8) for 30 min, and incubated with primary antibodies overnight at 4°C (E-cadherin, 3195, 1:100). After rewarming for 1 h at RT, cells were washed three times with PBS and stained with CY3-conjugated goat anti-rabbit antibody (Proteintech, SA00009-2, 1:200) for 1 h at RT in the dark. The antibody was aspirated, and 2 μg/ml of DAPI in PBS (YEASEN, 40728ES03) was added to stain for 5 min. Slides were rinsed three times with PBS and mounted with the Antifade Mounting Medium (Beyotime, P0126). Images were captured using a Nikon Eclipse 80i microscope.

## Supplementary Material

10.1242/biolopen.061790_sup1 Supplementary information
