# Supplementary material for: Constructing a doxycycline-inducible system for an epithelial-to-mesenchymal transition model in MCF10A cells
Source: Biol Open. 2024 Dec 9;13(12):bio061790. doi: 10.1242/bio.061790 (PMC11655024; doi:10.1242/bio.061790)
Supplement: Supplementary information [file biolopen-13-061790-s1.pdf]

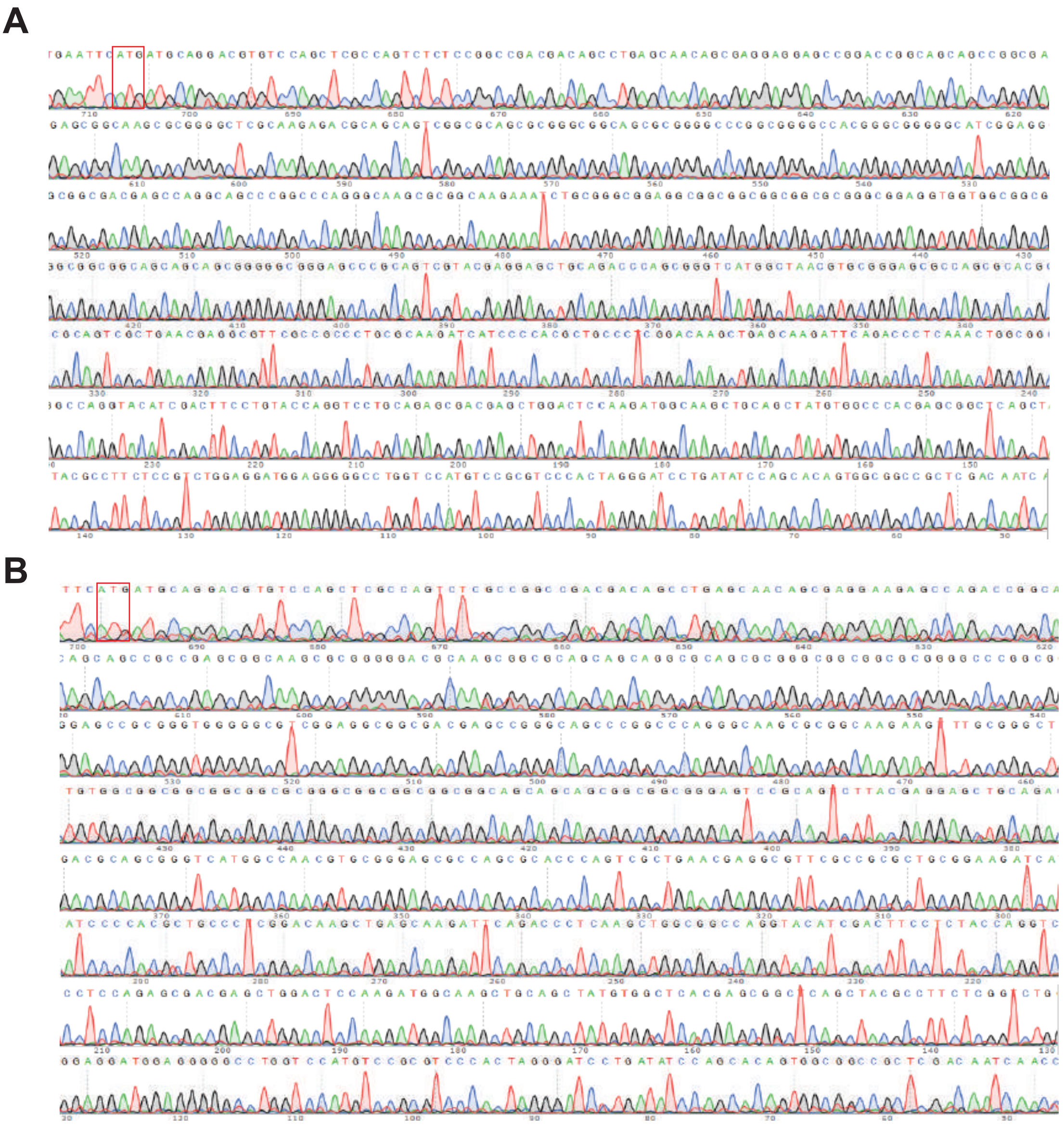

Fig. S1. Cloning of mTwist1 and hTWIST1 cDNA. (A) Sanger sequencing peaks for pLenti-mTwist1. (B) Sanger sequencing peaks for pLenti-hTWIST1. The start codons are indicated in red rectangles.

Biology Open • Supplementary information

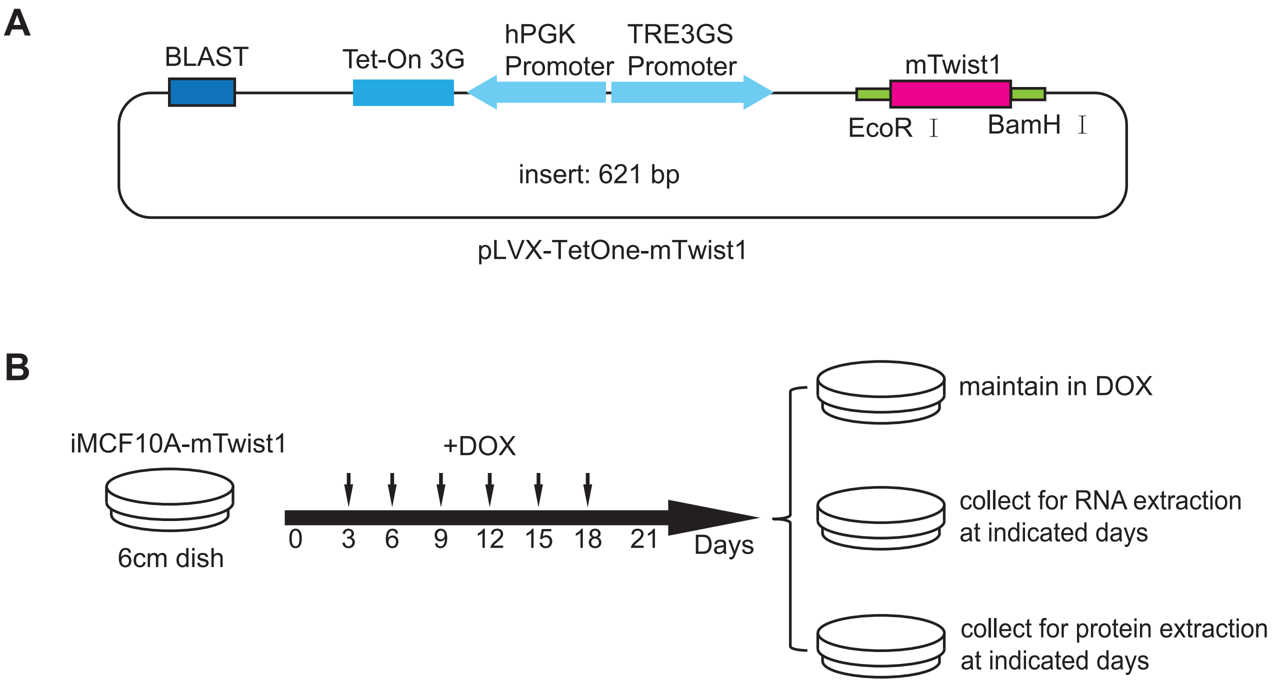

Fig. S2. Doxycycline induces a gradual EMT in MCF10A cells stably expressing pLVX-TetOne-mTwist1. (A) schematic diagram of pLVX-TetOne-mTwist1 plasmid. (B) Schematic overview of EMT induction in iMCF10A-mTwist1 cells.

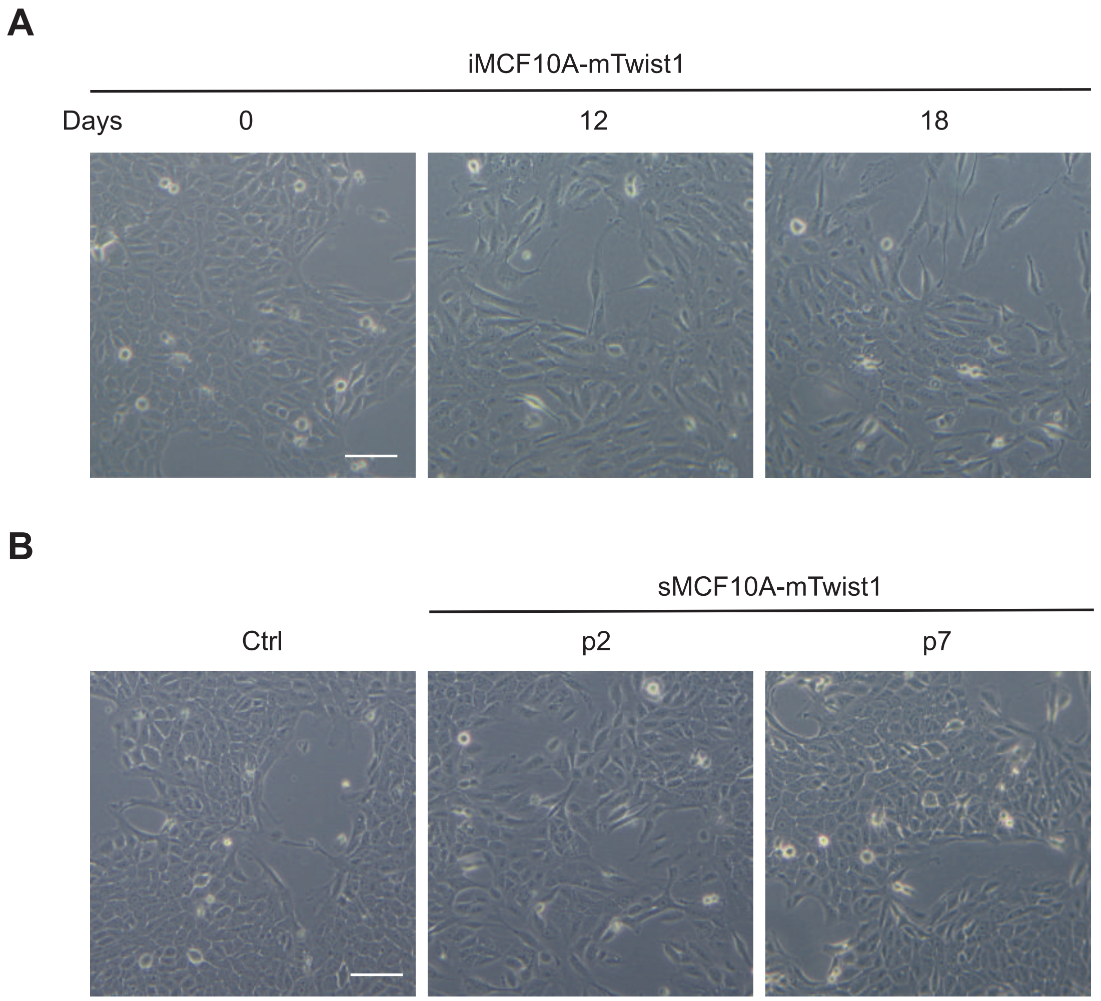

Fig. S3. The mesenchymal cell state can be reversed in polyclonal MCF10A cells expressing mTwist1**.** (A) Representa-tive Images showing morphological changes of iMCF10A-mTwist1 cells treated with DOX for 0, 12 and 18 days. (B) Representative Images showing the morphological changes of control (Ctrl) and sMCF10A-mTwist1 cells collected at passage no.2 (p2) and passage no.7 (p7). Scale bars: 100 µm.

Biology Open • Supplementary information

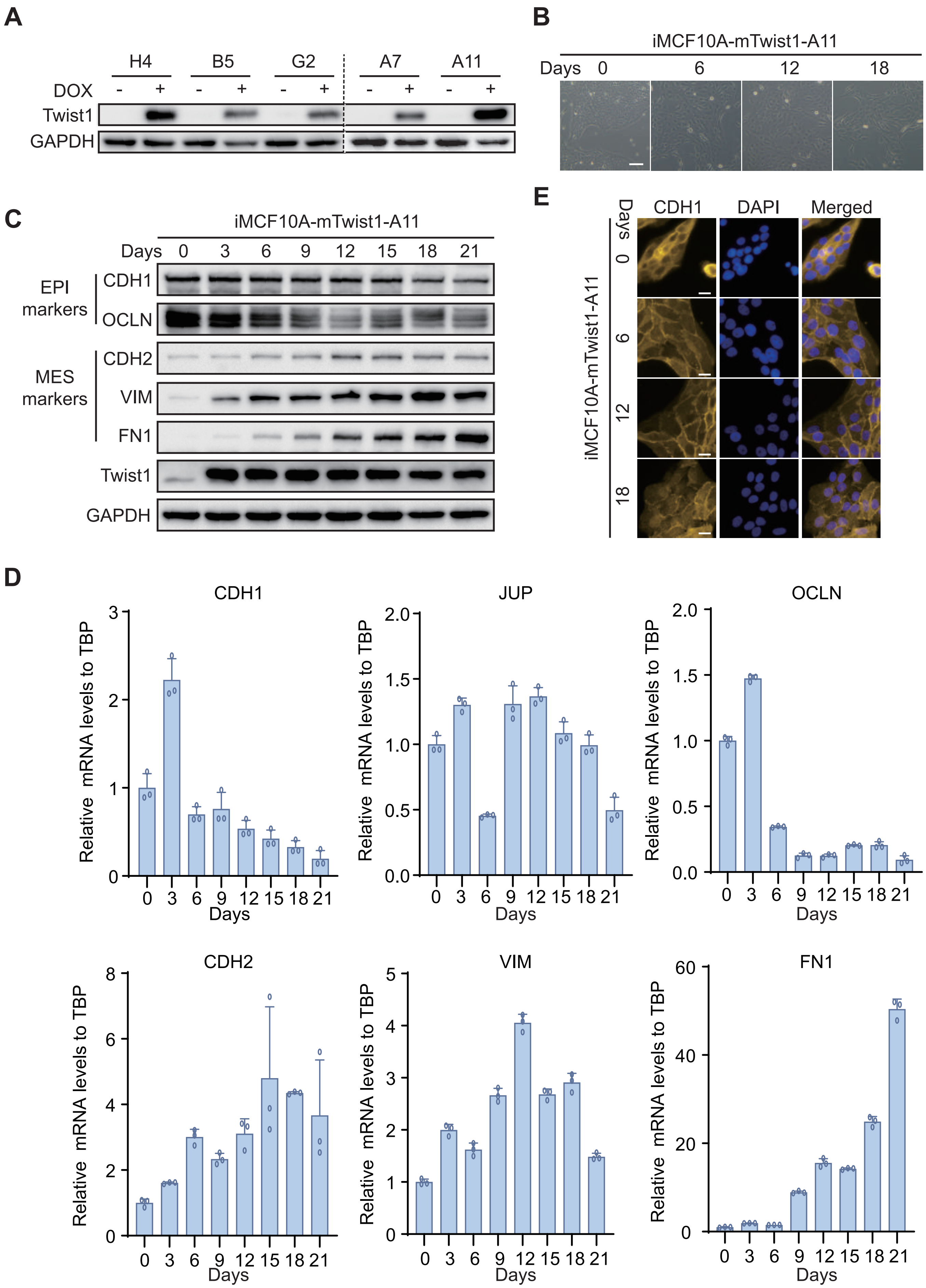
A
H4
B5
G2
A7
A11
DOX
- + - + - + - + - +
Twist1
GAPDH
B
iMCF10A-mTwist1-A11
Days 0 6 12 18
C
iMCF10A-mTwist1-A11
Days 0 3 6 9 12 15 18 21
EPI markers
CDH1
OCLN
MES markers
CDH2
VIM
FN1
Twist1
GAPDH
E
Days 0 6 12 18
iMCF10A-mTwist1-A11
CDH1
DAPI
Merged
D
CDH1
JUP
OCLN
CDH2
VIM
FN1
Relative mRNA levels to TBP
Days
0 3 6 9 12 15 18 21

Fig. S4. Monoclonal iMCF10A-mTwist1 cells show a stable epithelial-to-mesenchymal transition. (A) Expression of mTwist1 in monoclonal iMCF10A-mTwist1 cells, analyzed by Western blotting. (B) Representative Images showing morphological changes of monoclonal iMCF10A-mTwist1-A11 cells treated with DOX on days 0, 6,12, and 18. Scale bar: 100 µm. (C) Expression of EMT markers of clone A11, detected by Western blotting at indicated time points. (D) qRT-PCR analysis of of EMT marker expression of clone A11 at the same time points. Data are presented as mean ± SD, N=3. (E) Expression of CDH1 in clone A11, analyzed by immunofluorescence at indicated time points. Scale bar: 20 µm.

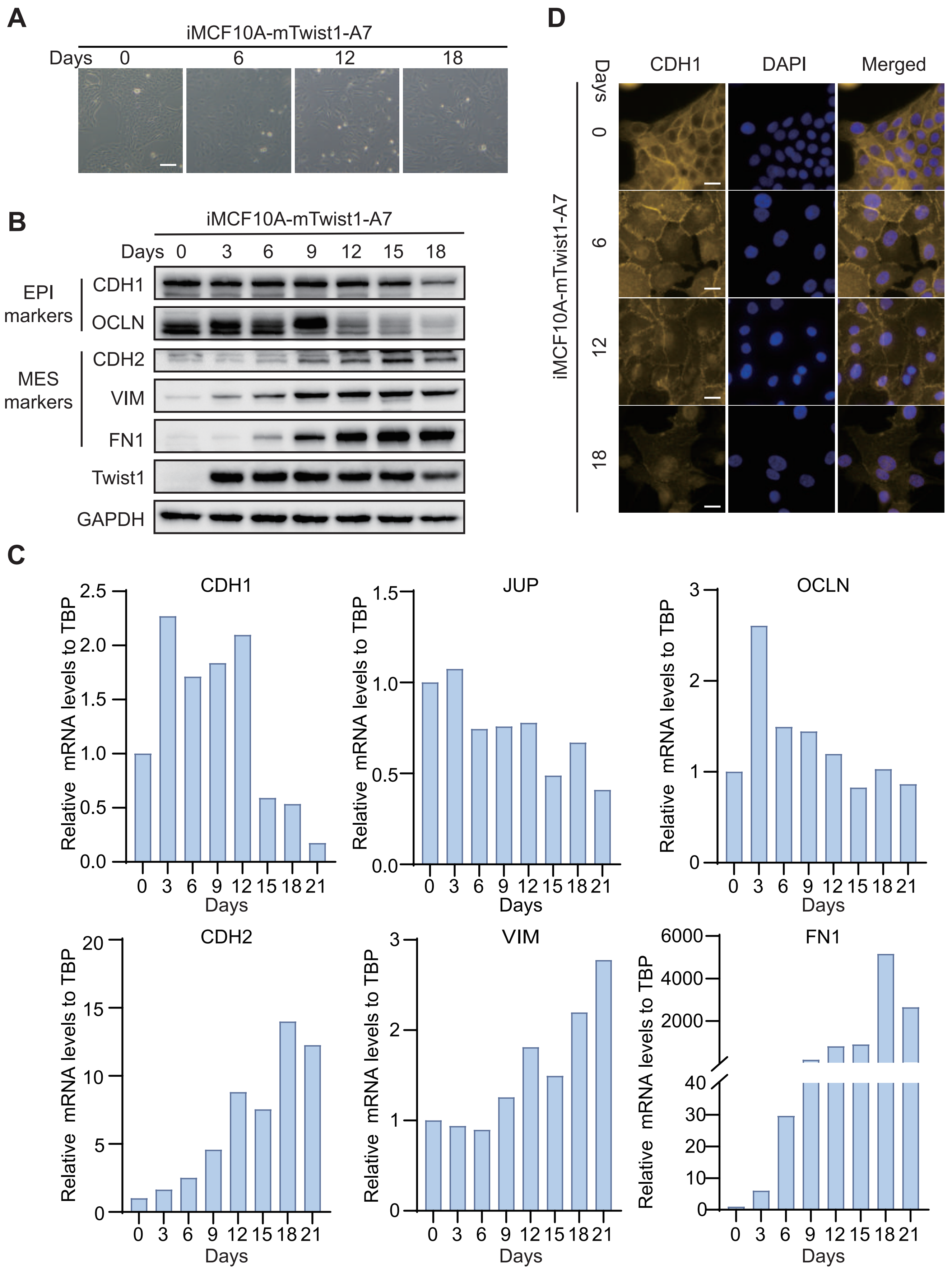

Fig. S5. Monoclonal iMCF10A-mTwist1 cells show a stable epithelial-to-mesenchymal transition. (A) Representative Images showing morphological changes of monoclonal iMCF10A-mTwist1-A7 cells treated with DOX on days 0, 6,12, and 18. Scale bar: 100 µm. (B) Expression of EMT markers of clone A7, detected by Western blotting at indicated time points. (C) qRT-PCR analysis of EMT marker epxression in clone H4 at the same time points. Data were from one time assay without error bars. (D) Expression of CDH1 in clone A7, analyzed by immunofluorescence at indicated time points. Scale bar: 20 µm.

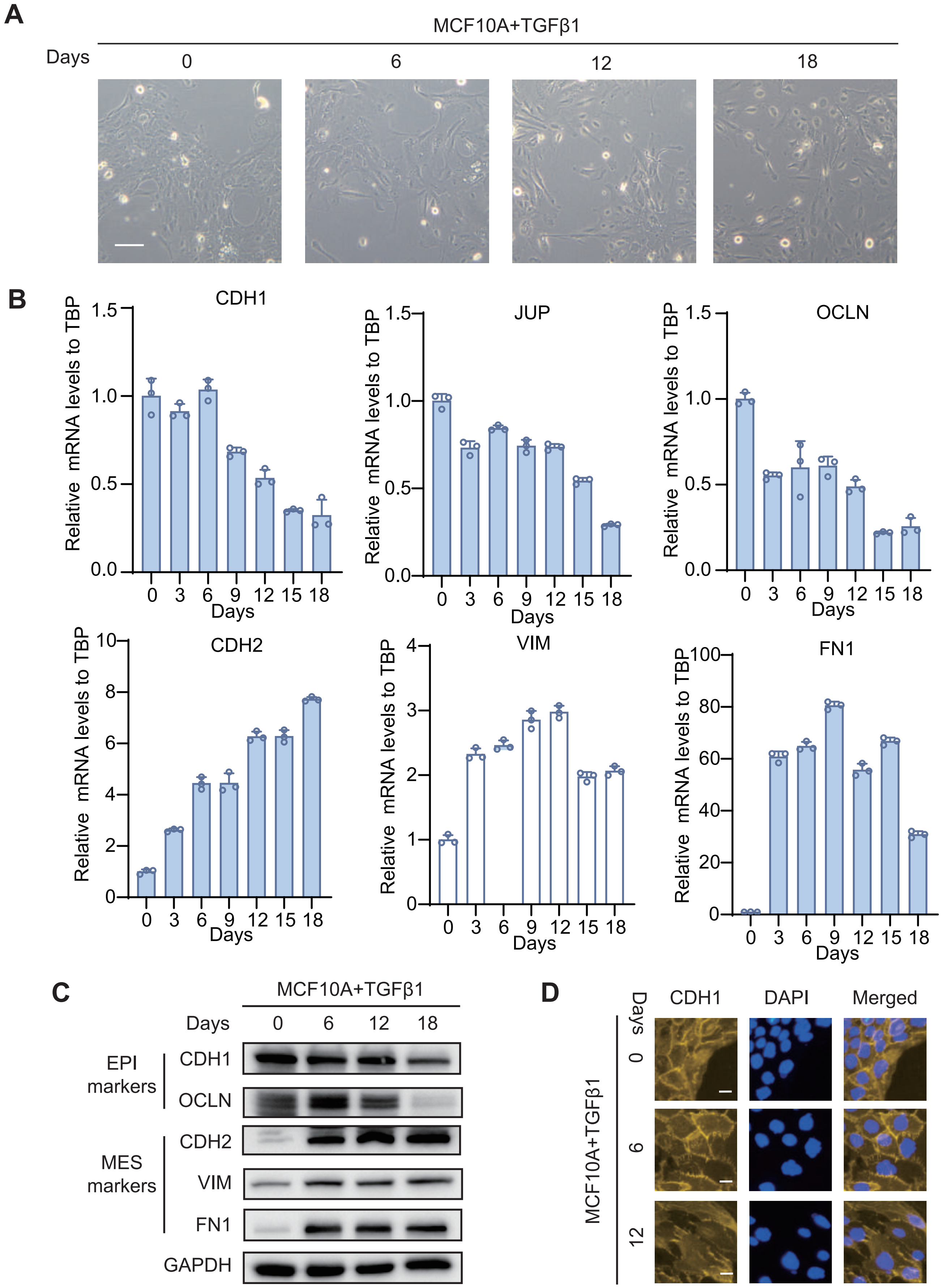
A
MCF10A+TGFβ1
Days
0
6
12
18
B
CDH1
JUP
OCLN
CDH2
VIM
FN1
Relative mRNA levels to TBP
Days
0 3 6 9 12 15 18
C
MCF10A+TGFβ1
Days 0 6 12 18
EPI markers
CDH1
OCLN
MES markers
CDH2
VIM
FN1
GAPDH
D
CDH1
DAPI
Merged
Days
MCF10A+TGFβ1
0
6
12

Fig. S6. TGFβ1 induces gradual EMT in MCF10A cells. (A) Representative Images showing morphological changes of MCF10A cells treated with 5 ng/mL TGFβ1 on days 0, 6,12 and 18. Scale bar: 100 μm. (B) Expression of EMT markers of TGFβ1 treated MCF10A cells, analyzed by qRT-PCR at indicated time points. Data are present-ed as mean ± SD, N=3. (C) Western blot detecting EMT marker expression at indicated time points. (D) Expression of CDH1, analyzed by immunofluorescence at indicated time points. Scale bar: 20 μm.

Table S1. Primer sequences of Twist1 cloning and qRT-PCR

Cloning	Primer Sequences (5'-3')
pLenti-TWIST1-forward	ACTCTAGTCCAGTGTGGTGAATTCATGATGCAGGACGTGTCCAGCTCGC
pLenti-TWIST1-reverse	CCACTGTGCTGGATATCAGGATCCCTAGTGGGACGCGGACATGGACCAGG
pLVX-TWIST1-forward	TCCTACCCTCGTAAAGAATTCATGATGCAGGACGTGTCCAGCTCGC
pLVX-TWIST1-reverse	CAGGGGAGGTGGTCTGGATCCCTAGTGGGACGCGGACATGGACCAG
qRT-PCR	
CDH1-forward	CAAAGTCACGCTGAATACAGTG
CDH1-reverse	GTCTTTGTTGGATTTGATCTGAACC
JUP-forward	AACCAGGAGAGCAAGCTGATC
JUP-reverse	ACAATGGCAGGCTTATTGCTG
OCLN-forward	ATTGTACTGGGGTTCATGATTATTG
OCLN-reverse	GCAGACACATTTTTAACCCACTC
CDH2-forward	CTGAGGAGTCAGTGAAGGAGTC
CDH2-reverse	ATCTCTATCAGACCTGATCCTGAC
VIM-forward	CTGCAATCTTTCAGACAGGATGTTG
VIM-reverse	GACATGCTGTTCCTGAATCTGAG
FN1-forward	CGAGAGTAAACCTGAAGCTGAAG
FN1-reverse	CCTTCATGGCAGCGGTTTG
TBP-forward	GTTCTGGGATTGTACCGCAGC
TBP-reverse	ACCGCAGCAAACCGCTTG

Table S2. Antibody information for Western blotting and immunofluorescence assay

Antibody	Company	Cat.No	Dilution ratio
**Western blotting**			
CDH1	Proteintech	20874-1-AP	1:1000
OCLN	Proteintech	13409-1-AP	1:2000
CDH2	Proteintech	66219-1-Ig	1:1000
VIM	Proteintech	60330-1-Ig	1:5000
FN1	Proteintech	15613-1-AP	1:2000
TWIST1	Proteintech	25465-1-AP	1:1000
GAPDH	Proteintech	60004-1-Ig	1:5000
HRP-conjugated Affinipure Goat Anti-Mouse IgG(H+L)	Proteintech	SA00001-1	1:10000
HRP-conjugated Affinipure Goat Anti-Rabbit IgG(H+L)	Proteintech	SA00001-2	1:10000
**Immunofluorescence assay**			
CDH1	Cell Signaling	3195	1:200
Cy3–conjugated Affinipure Goat Anti-Rabbit IgG(H+L)	Proteintech	SA00009-2	1:200

Biology Open • Supplementary information
